# Assessment of body composition in spinal cord injury: A scoping review

**DOI:** 10.1371/journal.pone.0251142

**Published:** 2021-05-07

**Authors:** Jan W. van der Scheer, Julia O. Totosy de Zepetnek, Cheri Blauwet, Katherine Brooke-Wavell, Terri Graham-Paulson, Amber N. Leonard, Nick Webborn, Victoria L. Goosey-Tolfrey

**Affiliations:** 1 Department of Public Health and Primary Care, THIS Institute, University of Cambridge, Cambridge, United Kingdom; 2 Peter Harrison Centre for Disability Sport, School of Sport, Exercise and Health Sciences, National Centre for Sport and Exercise Medicine, Loughborough University, London, United Kingdom; 3 Faculty of Kinesiology and Health Studies, University of Regina, Regina, SK, Canada; 4 Department of Physical Medicine and Rehabilitation, Spaulding Rehabilitation Hospital/Harvard Medical School, Boston, MA, United States of America; 5 International Paralympic Medical Committee, Bonn, Germany; 6 English Institute of Sport, Loughborough University, London, United Kingdom; 7 School of Sport and Service Management, University of Brighton, Brighton, United Kingdom; 8 British Paralympic Association, London, United Kingdom; University of Valencia, SPAIN

## Abstract

The objective of this scoping review was to map the evidence on measurement properties of body composition tools to assess whole-body and regional fat and fat-free mass in adults with SCI, and to identify research gaps in order to set future research priorities. Electronic databases of PubMed, EMBASE and the Cochrane library were searched up to April 2020. Included studies employed assessments related to whole-body or regional fat and/or fat-free mass and provided data to quantify measurement properties that involved adults with SCI. All searches and data extractions were conducted by two independent reviewers. The scoping review was designed and conducted together with an expert panel (n = 8) that represented research, clinical, nutritional and lived SCI experience. The panel collaboratively determined the scope and design of the review and interpreted its findings. Additionally, the expert panel reached out to their professional networks to gain further stakeholder feedback via interactive practitioner surveys and workshops with people with SCI. The research gaps identified by the review, together with discussions among the expert panel including consideration of the survey and workshop feedback, informed the formulation of future research priorities. A total of 42 eligible articles were identified (1,011 males and 143 females). The only tool supported by studies showing both acceptable test-retest reliability and convergent validity was whole-body dual-energy x-ray absorptiometry (DXA). The survey/workshop participants considered the measurement burden of DXA acceptable as long as it was reliable, valid and would do no harm (e.g. radiation, skin damage). Practitioners considered cost and accessibility of DXA major barriers in applied settings. The survey/workshop participants expressed a preference towards simple tools if they could be confident in their reliability and validity. This review suggests that future research should prioritize reliability and validity studies on: (1) DXA as a surrogate ‘gold standard’ tool to assess whole-body composition, regional fat and fat-free mass; and (2) skinfold thickness and waist circumference as practical low-cost tools to assess regional fat mass in persons with SCI, and (3) females to explore potential sex differences of body composition assessment tools.

**Registration review protocol:** CRD42018090187 (PROSPERO).

## Introduction

Individuals who suffer from neurologic trauma, such as spinal cord injury (SCI), undergo significant changes in body composition that increase the risk for secondary health conditions, including increased fat mass, decreased lean mass, and reduced bone density [1–5]. For example, the atrophy of metabolically active tissues and reduced activity levels result in increased risk of pressure ulcers, decreased energy expenditure, and a high risk of excess fat mass deposition under the skin as well as in viscera, liver and muscle [2, 6–9]. Reliable and valid body composition assessment tools are required to monitor profiles of fat and fat-free mass, and assess the effects of interventions (e.g., nutrition, exercise) to help improve these profiles [10, 11]. Understanding the reliability and validity of body composition assessment tools when applied for individuals with SCI will enable clinicians, researchers or other practitioners to make an informed decision regarding its use.

Body composition assessment tools range from simple to complex with all having limitations and some degree of measurement error [10, 12–14]. Examples include body mass index (BMI), waist circumference, skinfold thicknesses, bioelectrical impedance analysis (BIA), air displacement plethysmography (ADP), hydrostatic weighing, dual-energy X-ray absorptiometry (DXA), computed tomography (CT) and magnetic resonance imaging (MRI). Reliability and validity of these methods have often only be assessed in able-bodied populations, notwithstanding that many of these tools incorporate various assumptions that may or may not be met in people with a disability such as those with SCI, resulting in many interpretation difficulties [14].

As an example, a recent systematic review could only establish “low to moderate” confidence in the evidence showing that aerobic exercise can improve body composition in adults with chronic SCI [15], due to imprecision in the evidence. This imprecision may in part be explained by different body composition assessment tools employed, which may or may not be reliable and valid tools for adults with SCI. However, this cannot be confirmed due to a lack of SCI-specific information on the measurement properties of the employed tools.

We undertook a scoping review to address the lack of clarity on SCI-specific evidence for reliable and valid assessment of fat and fat-free mass, and help establish research priorities for SCI-specific body composition assessment. Scoping reviews, a type of knowledge synthesis, follow a systematic approach to map evidence on a topic and identify main concepts, theories, sources, and knowledge gaps [16–18]. We worked with an expert panel (including research, clinical, nutritional and lived SCI experience) on the design and interpretation of the review to ensure relevance of the results and help with identification of future research priorities [16, 19]. The review’s objectives were to: (1) map the evidence on measurement properties of tools to assess whole-body and regional fat and fat-free mass in adults with SCI; and (2) identify research gaps in order to set future research priorities.

## Methods

### Protocol and design

This scoping review protocol was developed using the methodological framework proposed by Arksey and O’Malley [16], and further enhanced by Levac et al. [20, 21]. This methodological framework includes the following steps: (1) identify the research question, (2) identify relevant studies, (3) study selection, (4) charting the data, (5) collating, summarizing and reporting the results, and 6) stakeholder consultation [20, 21]. The scoping review followed the relevant aspects of the Preferred Reporting Items for Systematic Review and Meta-Analysis Extension for Scoping Review Protocols (PRISMA-ScR) guidelines [18]. The review protocol was registered at the International Prospective Register of Systematic Reviews (PROSPERO) under the identification number CRD42018090187.

The scoping review was designed and conducted together with an expert panel (n = 8, Table 1), which included experts with lived SCI experience (paraplegia), a clinical (e.g. rehabilitation doctor) or other practitioner background (e.g. nutritionist), and/or a research background (i.e. expertise on SCI, body composition assessment, exercise physiology and/or performance nutrition). The panel collaboratively determined the scope and design of the review, and interpreted the findings [19]. This included three roundtable discussion meetings (in-person and online), and further exchange via email. Additionally, the expert panel reached out to their professional networks to gain further stakeholder feedback via interactive practitioner surveys and workshops with people with SCI. The goal of gaining this additional feedback was to benefit from a wider range of views that complemented those of the expert panel, without claiming this feedback represented all the views of the SCI population and various clinicians/practitioners working with people with SCI. The research gaps identified by the review, together with discussions among the expert panel including consideration of the survey and workshop feedback, informed the formulation of future research priorities.

**Table 1:** The expert panel (n = 8) that collaboratively determined the scope and design of the review, and interpreted its findings.

Expert panel member	Role/background
Blauwet, MD	Clinician
	Researcher
	Lived SCI experience
Brooke-Wavell, PhD	Researcher
Goosey-Tolfrey, PhD	Review team
	Researcher
Graham-Paulson, PhD	Nutritionist
	Researcher
Leonard, MSc	Review team
	Researcher
van der Scheer, PhD	Review team
	Researcher
Totosy de Zepetnek, PhD	Review team
	Nutritionist
	Researcher
Webborn, MD	Clinician
	Researcher
	Lived SCI experience

Note: Institutions and affiliations of each expert panel member can be found in the author section.

### Identification of the research question

Guided by international standards for reporting and developing clinical practice guidelines [22], the expert panel specified the target population (i.e., adults with SCI, in order to focus on a population in most need of guidance) and outcomes (i.e., fat and fat-free mass, considering already existing guidance on bone mass [23–25]). The following research objectives and outputs for this scoping review were specified:

1. Map the evidence on measurement properties (i.e., reliability, validity, responsiveness) of tools to assess whole-body and regional fat and fat-free mass in adults with SCI, and identify gaps in the evidence; and
2. Prioritize future research directions based on the identified gaps and expert panel discussion including the views of clinicians, researchers, other practitioners and people with SCI.

### Search strategy

PubMed, EMBASE (OVID) and the Cochrane library were searched for eligible studies up to April 1^st^, 2020. The search strategy was developed for PubMed and modified for the other databases (S1 File). Keywords were a combination representing three concepts: SCI (e.g., “spinal cord lesion”, “tetraplegia”, “paraplegia”, etc.); body composition assessment methods (e.g., “DXA”, “bioelectrical impedance”, “waist circumference”, etc.); and body composition outcomes related to fat and/or fat-free mass (e.g., “body weight”, “adipose tissue”, “lean mass”, etc.). Reference librarians verified the search strategy and the language was restricted to English, with case studies (N<3), unpublished studies, reviews and conference abstracts being excluded.

Eligible studies included an adult sample (≥16 years) where at least 50% of the participants were reported as traumatic or non-traumatic SCI (excluding multiple sclerosis and spina bifida), in line with a previous review (15). The focus for this search was on body composition assessment tools related to fat or fat-free mass (excluding mineral-only or water-only measures and measures of muscle morphology such as muscle fibre size and number of muscle fibres). Any body composition assessment method was eligible, including but not limited to: BMI, waist circumference, skinfold thicknesses, BIA, ADP, hydrostatic weighing, DXA, CT, MRI, and isotope dilution. Eligible study designs included statistics and/or individual data to quantify one or more of the following measurement properties:

- Test-retest reliability: Agreement of consecutive measurement(s) conducted under identical conditions [26].
- Intra-rater reliability: Agreement of consecutive measurement(s) conducted by the same investigator [26].
- Inter-rater reliability: Agreement between measurement(s) conducted by two different investigators [26].
- Criterion validity: Agreement of the method’s measure with the criterion ‘gold standard’ method, i.e., the 4-compartment model [26].
- Convergent validity: Agreement of the measure with ‘indirect’ measures (e.g. DXA, isotope dilution, MRI), but not to ‘doubly indirect’ methods (e.g. BIA, ultrasound, skinfold measurements) [26, 27]. Indirect refers to a method that assesses body composition using one estimate: e.g., an estimate of body fat % from the attenuation of two low-energy x-ray beams (DXA). Doubly indirect refers to a method that assesses body composition using two estimates: e.g., an estimate of body fat % from body density that was estimated from skinfold thicknesses.
- Responsiveness: The ability of the method to detect change over time when compared to the criterion and/or the convergent measure [26, 28].

### Study selection

**Fig 1:**
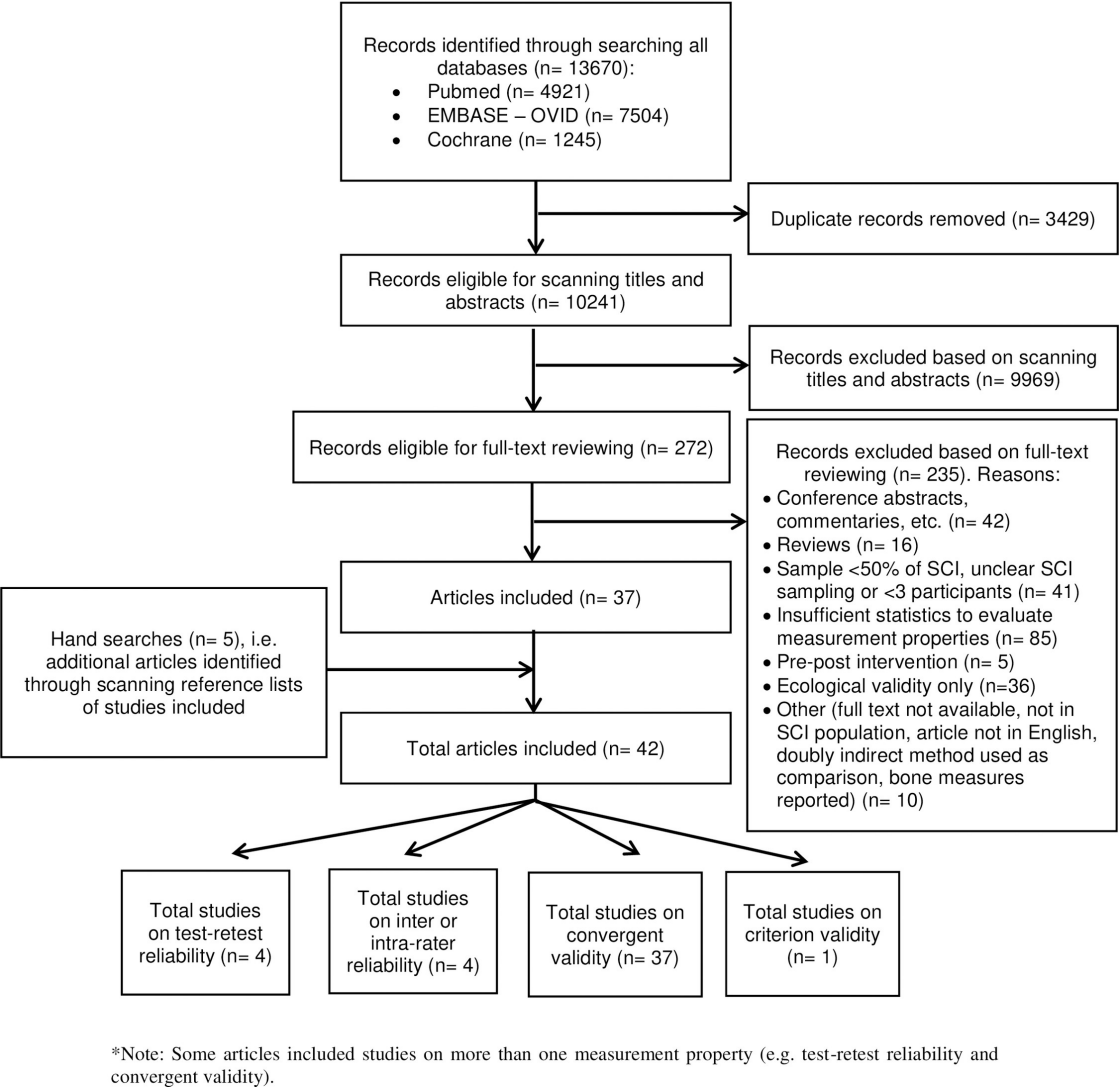
PRISMA flow diagram of the identification of eligible studies.

After duplicate removal, authors JTdeZ, AL and one other reviewer (see Acknowledgements) conducted title/abstract scanning and full-text screening (Fig 1). Each record and full text were judged independently by two reviewers (JTdeZ and AL); differences between the two reviewers were discussed and if necessary adjudicated by the first author JvdS.

### Data charting (extraction) and synthesis

Data charting was conducted by one reviewer and verified by another (JTdeZ and VG-T). Data charted from the eligible studies included participant demographics, study design and assessment tools used, and statistics/results. Each study was coded for showing “acceptable” reliability and/or validity or not, based on a minimum ICC of 0.70, Pearson’s *r* of 0.80, or equivalent [28, 29]. If a study’s reported ICC or *r* (or equivalent) was lower than 0.70 or 0.80, respectively, then “not acceptable” was used, while “inconclusive” denoted that a study’s reporting of statistical outcomes was incomplete.

**Fig 2:**
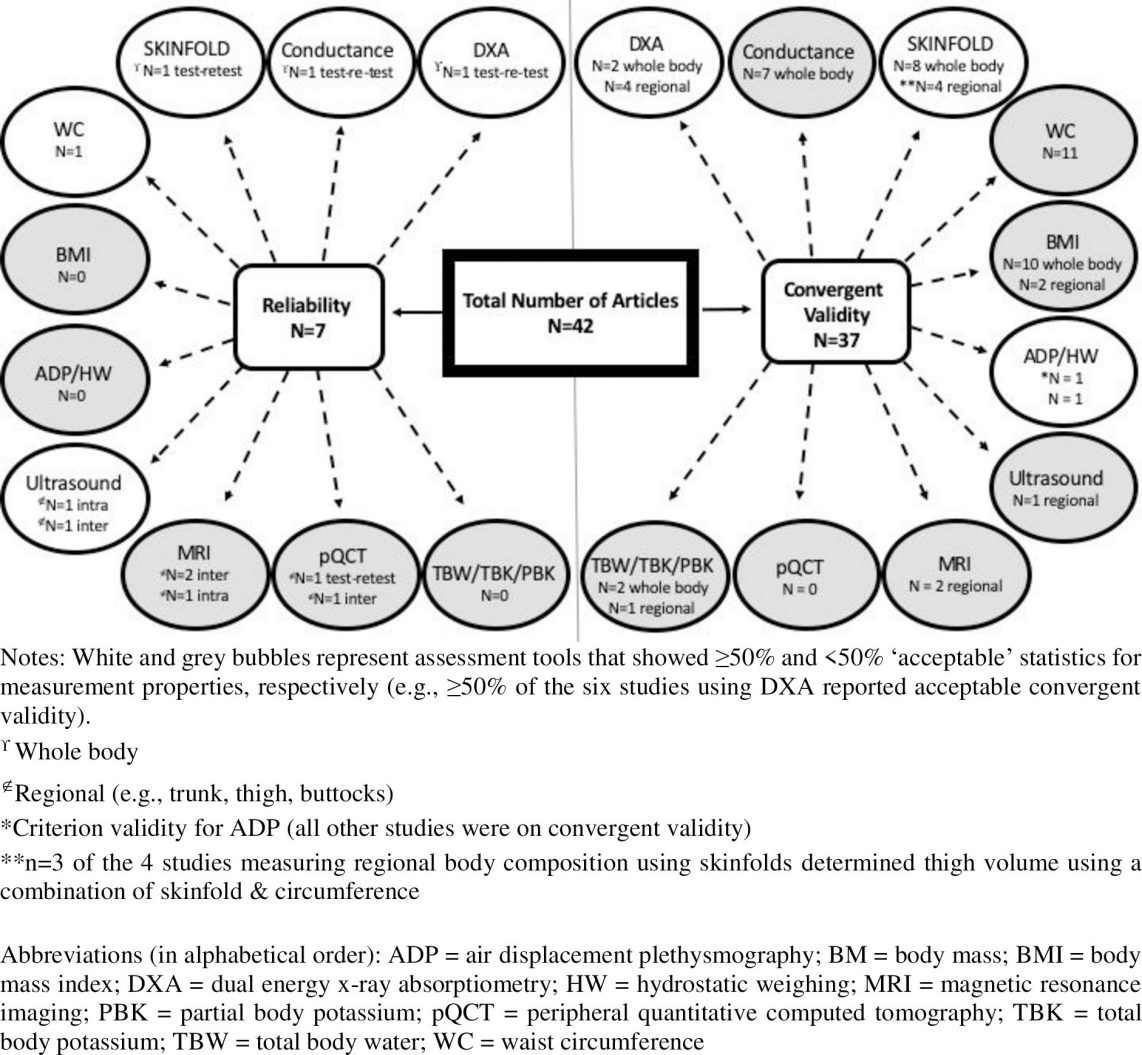
Mapping the available evidence: Total articles summarized by reliability and convergent validity.

**Fig 3:**
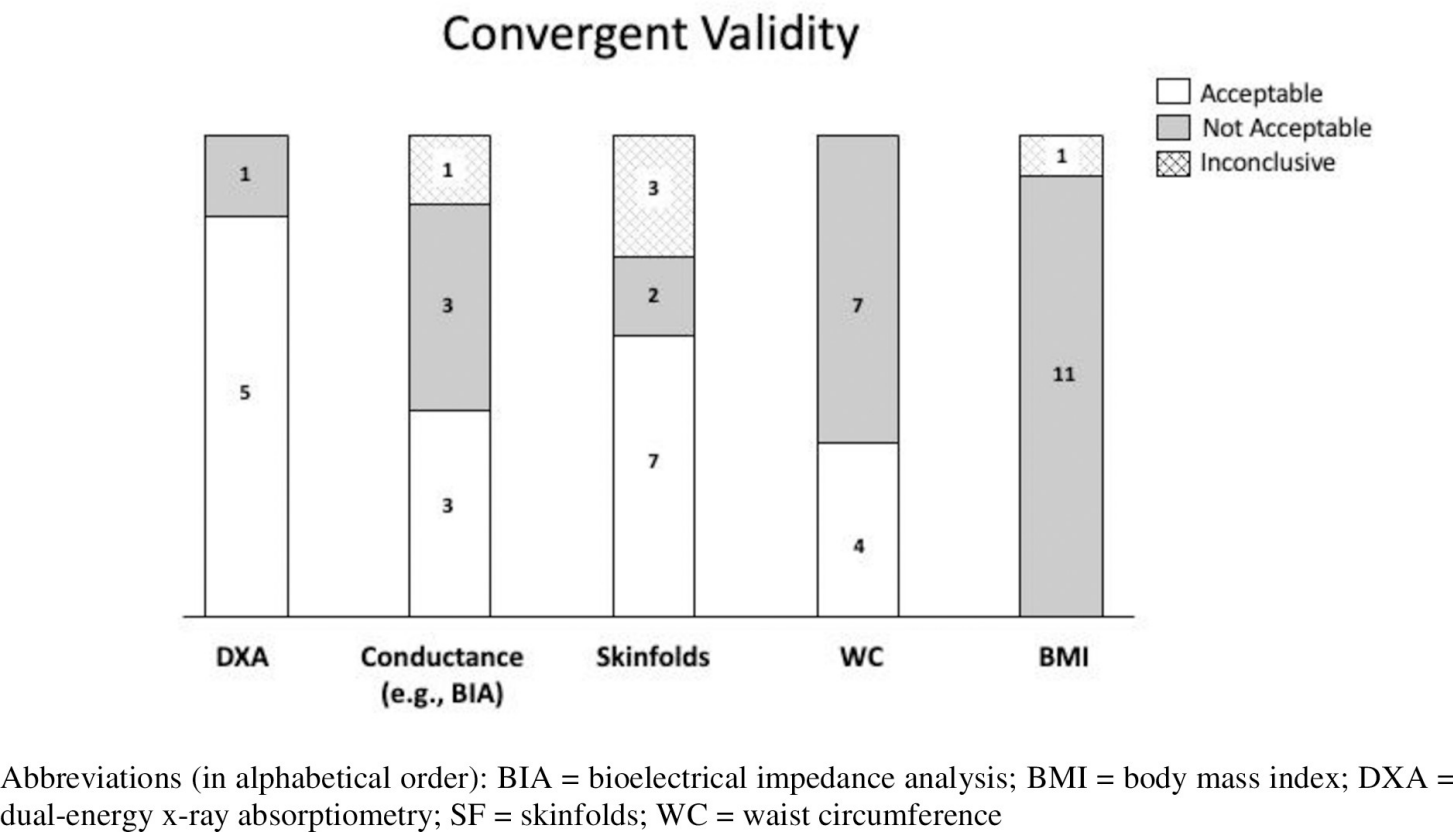
Number of studies with “acceptable” convergent validity (e.g. reported ICC > 0.70), that were “not acceptable” (e.g. reported ICC < 0.70), or “inconclusive” (e.g. reported statistics incomplete) for each of the most commonly evaluated assessment tools (whole-body and regional studies combined).

Maps of the evidence were created using the data charted from the eligible studies (Figs 2 and 3). It provides a visual overview of the tools used in studies on reliability and/or validity, recognising that any measurement tool should be both reliable (e.g., test-retest reliability) and valid (e.g., at least convergent validity if not criterion validity) in order to recommend its use in research or clinical practice [30].

### Stakeholder consultation

As described above under “Protocol and design”, the expert panel used a convenience sample approach (i.e. reaching out to their professional networks) to benefit from a wider range of professional and SCI views that complemented those of the expert panel. Authors CB and JvdS hosted two small group workshops that included five people with lived SCI experience (three men and two women with paraplegia [n = 3] or tetraplegia [n = 2] for longer than one year) at Spaulding Rehabilitation Hospital (Boston, MA, USA). These participants drew on their own experience living with SCI as well as that of others, given that participants were selected for their connections to a large peer network. All participants had undergone body composition measurements as a part of their rehabilitation process in either a home, clinical, or sports setting. During the workshop, participants discussed with the hosts their views on the importance of measuring body composition, their preferences of and experiences with different tools, and barriers they had experienced while using the tools. These views, preferences and experiences were captured and synthesised qualitatively (see S2 File).

Authors VG-T, TG-P and JvdS facilitated two interactive surveys with 15 clinicians and other practitioners working in the field of body composition and SCI, with competencies as nutritional, elite sport, sport science, and/or clinical research practitioners. The surveys were conducted in group sessions at Loughborough University (UK) and Spaulding Rehabilitation Hospital (USA). On-line and live surveys combined with group discussions were used to gain the views, preferences, and experienced/perceived barriers with different body composition assessment tools. Responses were synthesised qualitatively (see S3 File).

For the surveys and workshops, after being informed about the project and anticipated use of their data, all participants provided verbal informed consent. Ethical clearance was obtained from the Loughborough University ethics advisory committee (human participants subcommittee; UK).

## Results

Out of 13,670 identified titles, a total of 42 eligible studies [8, 31–71] were identified: the bibliographic database search provided 37 articles that met the eligibility criteria, and an additional five articles were identified after scanning reference lists of articles included (Fig 1). Common reasons for exclusion were insufficient data or statistics to evaluate measurement properties, conference abstracts or commentaries only, and insufficient or unclear SCI sampling (e.g. <50% or not reporting the proportion of people with SCI as part of the total sample). Data charting details for each of the 42 eligible studies are provided in Table 2.

**Table 2:** Data charting (extraction) of eligible studies in alphabetical order by first author.

Author/Date	N (M/F)	Participants Characteristics mean±SD (range)	Body Composition Assessment Tools	Statistics (Results)
Beck et al., 2014 [31]	13 (7/6)	Age: 41.5±7.8 (25–50) TSI: 11.7±9.1 (2–26) AIS: A-B NLI: para (T3-T12)	• BMI: mass measured on scale; height self-report • DXA (GE Lunar Prodigy): TB fat%	• **Convergent validity of BMI (vs. DXA)** **not acceptable:** BMI underestimated body fat in males and females with SCI
Buchholz et al., 2003 [32]	31 (19/12) *includes n = 7 spina bifida	Age: 34.2±8.8 (20–57) TSI: 13.8±11.8 years AIS: 21 A-B; 10 C-D NLI: para	• BMI: mass measured on scale, length measured supine with adult-sized Plexiglas length board • BIA: frequencies of 5, 50, and 200kHz to predict TBW & ECW • TBW (99.9% ^2^H_2_O): 0.25g/kg body mass; blood pre and post • ECW (3% NaBr): 1mL/kg body mass; blood pre and post	• **Convergent validity of BMI (vs. TBW)** **inconclusive:** poor sensitivity (20%) • **Convergent validity of BIA at 50kH (vs. TBW)** **acceptable**: R^2^ = 0.91–0.96 • **Convergent validity of BIA at 50 kHz (vs. ECW)** **acceptable**: R^2^ = 0.66
Bulbulian et al., 1987 [33]	22 (22/0) *wheelchair athletes	Age: 27.5±5.9 TSI: NR AIS: A-B NLI: para (T1-L2)	• Anthropometrics: dominant side for diameters (cm), circumferences (cm), skinfolds (mm, Harpenden calipers) to predict D_b_ • HW: Behnke & Wilmore protocol to determine V_b_ [corrected for residual lung volume via closed-circuit O_2_ dilution]; D_b_ and %fat calculated	• **Test re-test reliability diameters, skinfolds, circumferences** **acceptable:** r = 0.97–0.99 • **Convergent validity of anthropometrics (vs. HW)** **inconclusive**: ○ Diameters: r = 0.60 ○ Skinfolds & circumferences: r = 0.95
Cirnigliarro et al. 2013 [34]	30 (29/1)	**TETRA (n = 14)** Age: 45±8 TSI: 20±14 AIS: 7 A-B; 7 C-D **PARA (n = 16)** Age: 41±13 TSI: 9±11 AIS: 13 A-B; 3 C-D	• BIS: 256 frequencies ranging from 3–1000 kHz to predict ECV & ICV in TB and legs/arms • DXA (GE Lunar iDXA): TB & legs/arms LM	• **Convergent validity of BIS ECV & ICV (vs. DXA TB LM)** **acceptable for tetra only**: ○ ECV para r = 0.75, tetra r = 0.89 ○ ICV para r = 0.72, tetra r = 0.88 • **Convergent validity of BIS ECV & ICV (vs. DXA legs LM)** **acceptable for ECV only**: ○ ECV para r = 0.86–0.87, tetra r = 0.81–0.88 ○ ICV para r = 0.79–0.92, tetra r = 0.76–0.78 • **Convergent validity of BIS ECV & ICV (vs. DXA arms LM)** **not acceptable**: ○ ECV para r = 0.0.54–0.55, tetra r = 0.48–0.92 ○ ICV para r = 0.0.42–0.46, tetra r = 0.0.16–0.44
Cirnigliaro et al., 2015 [35]	63 (63/0)	Age: 40.0±7.2 TSI: 16.1±12.7 AIS: 29 A-B; 34 C NLI: 33 para; 30 tetra	• BMI: mass and length (electronic calipers) determined from DXA • WC: midpoint btw top iliac crest and last rib at end expiration with flexible measuring tape • DXA (GE Lunar iDXA): VAT_vol_	• **Convergent validity of BMI (vs. DXA VAT**_**vol**_**)** **not acceptable:** r = 0.59–0.67 • **Convergent validity of WC (vs. DXA VAT**_**vol**_**)** **not acceptable:** r = 0.59–0.66
Clasey et al., 2005 [36]	20 (14/6)	Age: 36.1±10.5 (18.5–56.4) TSI: 10.2±9.5 AIS: 20 A-B NLI: para (T3-L2)	• ADP (BodPod): V_b_ measured [thoracic volume obtained]; D_b_ calculated • HW: V_b_ measured [corrected for residual lung volume via O_2_ dilution technique]; D_b_ calculated • 4-comp model (13/20 participants via Heymsfiel et al.): D_b_ from ADP & HW, TBW from D_2_O [blood pre and post], TB bone and TB mass from DXA [Lunar DPX-IQ software version 4.3]	• **Criterion validity of HW/ADP (vs. 4-comp model)** **acceptable**: ○ Shared variance between HW/ADP and 4-comp model in persons with injuries below T3; must obtain thoracic volume
Cragg et al., 2015 [37]	27 (19/8)	Age: 40±11 TSI: 14±10 AIS: 20 A-B; 7 C-D NLI: 11 para; 16 tetra	• BMI: mass determined from DXA, length from either self-report or electric ruler on DXA • WC: supine at narrowest part of waist at end expiration • DXA (Hologic QDR 450): TB fat [kg & %], abdominal fat [kg & %]	• **Convergent validity of BMI (vs. DXA TB)** **acceptable** **for mass [kg]:** r = 0.90 ○ Caveat: no analysis of % body fat and BMI to evaluate whether BMI underestimates obesity • **Convergent validity of WC (vs. DXA abdominal)** **acceptable for mass [kg] only**: % r = 0.76; kg r = 0.82
Desport et al., 2000 [38]	20 (15/5)	Age: 45.4±12.8 TSI: >4mo AIS: NR NLI: 15 para; 5 tetra	• BIA: frequencies of 50 & 100kHz to predict TBW • Skinfolds: triceps, biceps, subscap, suprailiac (Harpenden calipers & Durnin’s technique) to predict D_b_ & TB fat% • TBW (2% ^18^O): 15mg/kg body mass; saliva pre and post	• **Convergent validity of BIA (vs. TBW)** **inconclusive**: Bland-Altman showed good agreement using 100kHz **Convergent validity of skinfold (vs. TBW)** **inconclusive**: triceps site most accurate
Edwards et al., 2008 [8]	15 (12/3)	Age: 39.8±7.4 (28–49) TSI: 16.5±8.7 (1.1–28.7) AIS: 11 A-B; 4 C-D NLI: 6 para; 9 tetra	• WC: 3 sites supine: 1) immediately below lowest rib; 2) immediately above iliac crest; 3) midpoint btw lowest rib and iliac crest, all at end expiration • CT (GE): total, SAT, VAT from single slice scan btw L4-L5	• **Test-retest reliability of WC** **acceptable at all 3 sites**: ICC = 0.998–0.999 • **Convergent validity of WC (vs. CT)** **acceptable at all 3 sites**: r = 0.91–0.93
Emmons et al., 2011 [39]	24 (24/0)	Age: 39±11 (23–64) TSI: 19±11 (2–36) AIS: 19 A-B; 5 C-D NLI: 8 para; 16 tetra	• Ultrasound (GE): supine using 2–5 MHz curvilinear transducer to measure SAT & VAT • DXA (GE Lunar Prodigy): TB fat %, trunk fat (TRK%), android (A%) and waist fat (W%)	• **Convergent validity of ultrasound for SAT (vs. DXA)** **not acceptable:** r = 0.29–0.39 • **Convergent validity of ultrasound for VAT (vs. DXA)** **not acceptable:** r = 0.28–0.42
George et al. 1988 [40]	15 (10/5)	Age: 30.8 ± 7.9 TSI: 7.9±6.3 (0.8–23.5) AIS: 8 A-B; 7 C-D NLI: 11 para, 4 tetra	• TBW (ethanol dilution): 0.35g/kg body mass; breath analysis pre and post; to predict FFM HW: Behnke & Wilmore protocol to determine V_b_ [corrected for residual lung volume helium dilution]; D_b_ and TB fat% calculated	• **Convergent validity of TBW for FFM (vs. HW)** **not acceptable**: r = 0.71
Goosey-Tolfrey et al., 2016 [41]	30 (30/0) *wheelchair athletes	Age: 30.8 ± 7.9 TSI: 7.9±6.3 (0.8–23.5) AIS: 8 A-B; 7 C-D NLI: 11 para, 11 tetra; 8 other (diastrophic dysplasia, hip damage, amputation)	• Skinfolds: biceps, triceps, subscapular, iliac crest, supraspinale, abdominal, front thigh, medial calf (Harpenden calipders & several prediction equations) to predict D_b_ & TB fat% • BIA (Bodystat 1500): single frequency 50 kHz & Lukaski prediction equation for TB fat% • ADP (BodPod): V_b_ measured [thoracic volume estimated via Dempster & Aitken]; D_b_ and TB fat% calculated • DXA (GE Lunar Prodigy Advance): TB fat (kg, %), TB LM, FFM	• **Convergent validity of skinfolds, BIA, ADP (vs. DXA fat%)** **not acceptable**: all ICCs were <0.7
Gorgey et al. 2011 [44]	13 (13/0)	Age: 35±8 (22–45) TSI: 12±8 (2–19) AIS: A-B NLI: 6 para, 7 tetra	• WC: seated at level of narrowest part of torso at end expiration using inelastic tape • MRI: 1.5 or 3T whole body scanner (GE Signa) for multi-axial slices of abdomen; determine SAT & VAT CSA and volume (Win Vessel 2 software); analyzed by 2 examiners • DXA (Lunar Prodigy Advance & Hologic QDR-2000 scanner) for TB FM; measured 2x	• **Inter-rater reliability for CSA of MRI slices (2 examiners)** **inconclusive**: VAT 13% error; SAT 1.5% error • **Intra-rater reliability for CSA of MRI slices (1 image analyzed 2x)** **inconclusive**: VAT 3%; SAT 0.5% • **Convergent validity of WC (vs. MRI SAT & VAT)** **not acceptable:** r = 0.67–0.74 • **Convergent validity of single slice MRI at L4-L5 (vs. DXA)** **not acceptable**: r = 0.7–0.76
Gorgey et al. 2012 [43]	63 (63/0)	Age: 41±11 (18–65) TSI: 14±10 (0.9–65) AIS: A-B NLI: 48 para;15 tetra	• Body mass: measured on wheelchair scale • DXA (Lunar Prodigy Advance): legs, trunk, TB FM	• **Convergent validity of body mass to predict legs, trunk, TB FFM (vs. DXA)** **not acceptable**: legs R^2^ = 0.25; trunk R^2^ = 0.56; TB R^2^ = 0.53
Gorgey et al. 2018 [42]	Short Term: 24 (24/0) Long Term: 22 (22/0)	**Short-term:** Age: 38.5±10 (19–57) TSI: 9±9.5 (1.5–31) AIS: 18 A-B; 6 C-D NLI: 8 para; 16 tetra **Long-term:** Age: 36±10 (18–49) TSI: 8±8 (1.3–28) AIS: 22 A-B NLI: 13 para; 9 tetra	• DXA (GE Lunar Prodigy Advance): TB fat (kg, %), FFM, LM; regional (trunk, legs, arms, android, gynoid) fat (kg, %), FFM, LM; measured 2x by 1 technician	• **Short-term test-retest reliability of DXA** **acceptable**: CV for TB and regional measures between 2.3–6.5% • **Long-term test-retest reliability of DXA** **acceptable (except for android region %fat)**: CV for TB and regional measures between 2–6%; ICC for TB and regional measures >0.97
Inayama et al. 2014 [45]	74 (74/0)	Age: 45.6±14.3 (33.8–59.3) TSI: 14.6±10.3 (5.3–21.6) AIS: NR NLI: 32 para; 42 tetra	• BMI: mass measured on wheelchair scale; length measured supine using inelastic tape measure • WC: supine at the level of umbilicus at end expiration • CT scanner (HiSpeed Advantage, GE): 120 kVp & 400 mAs for measurement of VAT	• **Convergent validity of BMI (vs. CT)** **not acceptable**: R^2^ = 0.439 • **Convergent validity of WC (vs. CT)** **not acceptable**: R^2^ = 0.604
Jones et al. 2003 [46]	19 (19/0)	Age: 45.6±14.3 (16–52) TSI: 14.6±10.3 (5.3–21.6) AIS: 16 A-B; 3 C-D NLI: 7 para; 13 tetra	• BMI: mass determined from from DXA; height self-reported • DXA (Lunar DPX-L): TB FM	• **Convergent validity of BMI (vs. DXA)** **not acceptable**: BMI underestimated body fat in males with SCI
Layec et al. 2014 [47]	8 (6/2)	Age: 42 ± 8 TSI: 9 ± 3 (4–16) AIS: A NLI: para (T6–T12)	• Anthropometrics: thigh and lower leg volume via circumferences, length, and skinfold thickness • ^1^H-MRI: 3T (Tim-Trio, Siemens Medical Solutions), turbo spin echo sequence to obtain 15–20 trans-axial images of thigh & lower leg	• **Convergent validity of leg volume (vs. MRI)** **acceptable:** thigh R^2^ = 0.89; lower leg R^2^ = 0.98; however, Bland-Altman showed slight systematic over-estimation of muscle volume
Lester et al. 2019 [48]	32 (31/1)	Age: 37±11 TSI: 10±10 AIS: A-C NLI: 22 para; 10 tetra	• DXA (Lunar iDXA): thigh LM • MRI: 1.5T (GE Signa), 12–15 trans-axial images from hip to knee	• **Convergent validity of DXA thigh LM (vs. MRI)** **acceptable**: R^2^ = 0.90; Bland-Altman showed slight systematic overestimation of thigh LM
Maggioni et al. 2003 [49]	13 (13/0)	Age: 33.8±5.4 TSI: 13.9±5.8 AIS: NR NLI: 12 para; 1 tetra	• Skinfolds: biceps, triceps, suprailiac and subscapular (Holtain calipers and Durnin-Womersley equation) to predict D_b_ & TB fat% • DXA (Lunar DPX-IQ): TB fat & fat-free (kg, %)	• **Convergent validity of skinfolds (vs. DXA)** **inconclusive**: no stats reported: “fat mass measured with skinfold method was significantly lower compared to DXA”
McCauley et al. 2018 [51]	22 (22/0)	Age: 37±10.3 (18–50) TSI: 8.3±7.8 (1–28) AIS: NR NLI: 14 para; 8 tetra	• Anthropometrics: WC (level of umbilicus in supine position at end expiration with an inflexible measuring tape) and skinfolds (abdominal & suprailiac using (Harpenden calipers) • MRI: 1.5T (GE Singa), transverse axial images of trunk region for determination of SAT and VAT	• **Convergent validity of anthropometrics (vs. MRI)** **acceptable**: SAT R^2^ = 0.76; VAT R^2^ = 0.72
McCauley et al. 2020 [50]	27 (27/0)	Age: 38.5±10.5 (18–61) TSI: 10.3±9.8 (1–29) AIS: 26 A-B; 1 C NLI: C5-L1	• DXA (GE Lunar iDXA): android regions for VAT_mass_ (encore software) • MRI: 1.5T (GE Singa): multiaxial images of trunk region for determination of SAT and VAT	• **Convergent validity of DXA VAT (vs. MRI)** **acceptable**: SAT R^2^ = 0.82; VAT R^2^ = 0.92
Mesbah et al., 2019 [52[	16 (13/3)	Age: 32.4±9.1 TSI: 6.7±7.7 AIS: 15 A-B; 1 C NLI: NR	• MRI: 3T (Siemens): 50 images of thigh [between greater trochanter and lateral epicondyle of femur] analyzed for SAT, IMAT, and lean tissue; images obtained and analyzed via novel fully automated and manual volumetric segmentation	• **Convergent validity of fully automated analysis (vs. manual analysis)** **acceptable:** precision = 0.98–1 for SCI and 0.82–1 for able-bodied
Modlesky et al. 2004 [53]	8 (8/0)	Age: 35±9 TSI: > 2 yr AIS: AIS A-B NLI: C6-L1	• DXA (Hologic, Delphi A): leg FFM • MRI: 1.5T (GE): 25 axial images of thigh analyzed for LM	• **Convergent validity of DXA thigh FFM (vs. MRI thigh LM)** **acceptable**: r = 0.99
Mojtahedi et al. 2009 [54]	16 (8/8) *wheelchair athletes	**Female**: Age: 22.0±2.7 (18–27) TSI: 16.9±4.1 (13–24) AIS: A-B NLI: para (T5-L5) **Male**: Age: 21.9±4.2 (19–31) TSI: 15.4±7.3 (4–25) AIS: 3 A-B; 5 C-D NLI: para (T5-L5)	• Skinfolds: triceps, subscapular, biceps, chest, midaxillary, paraumbilical, suprailiac, thigh and lateral calf (Harpenden calipers and Jackson & Pollock protocol) to predict D_b_ & TB fat% • BIA (RJL Systems Analyzer; Quantum X) to predict TB fat% • DXA (Hologic QDR 4500A): TB fat%	• **Convergent validity of skinfold (vs. DXA)** **acceptable for men only**: females r = 0.63–0.81; males r = 0.84–0.97 • **Convergent validity of BIA (vs. DXA)** **not acceptable:** females r = 0.48–0.57; men r = 0.55–0.73
Olle et al. 1993 [55]	17 (17/0)	Age: 32.4±5.6 (23–43) TSI: >3 years NLI: C6-L12 AIS: NR **active group (n = 12); sedentary group (n = 5)	• TOBEC: supine using 2.5MHz electromagnetic field to estimate FFM; measured 2x by 1 technician • Skinfolds: sum of 7 abdominal, anterior thigh, biceps, chest, subscap, suprailiac, triceps taken seated (Lange calipers)	• **Test-retest reliability (within day) of TOBEC** **acceptable**: ICC = 0.994–0.999 • **Convergent validity of skinfolds (vs. TOBEC)** **not acceptable**: r = 0.73
Panisset et al. 2018 [56]	20 (18/2)	Age: 42.5 (18–82) TSI: 41 days (17–75 days) AIS: A-D NLI: C1-L5 (16 Tetra, 4 Para)	• BIA (model SFB7, ImpdiMed Ltd): 256 frequencies ranging from 3–1000 kHz (7 prediction equations) to predict TBW & TB FFM • DXA (GE-Lunar Prodigy): TB FM and FFM • TBW (99.9% ^2^H_2_O and 97% ^18^O): 0.2g/kg body mass; urine pre and post	• **Convergent validity of BIA (vs. TBW): most equations** **acceptable**: r = 0.12–0.94; Bland-Altman show some equations over- and some under-estimate FFM (best was BIA_K50_) • **Convergent validity of DXA (vs. TBW)** **acceptable:** r = 0.88; however, Bland-Altman showed systematic under-estimation of FFM of 1.7kg (2.9%)
Pelletier et al. 2016 [57]	136 (100/36)	Age: 49.1±12.9 TSI: 15.6±11.3 AIS: A-D NLI: 70 para; 66 tetra	• BMI: mass measured on wheelchair scale; height self-reported • WC: supine position at level of lowest rib • DXA (Hologic Discovery QDR 45000W): VAT (cm^2^), Trunk fat (kg, %), TB fat (kg, %)	• **Convergent validity of BMI (vs. DXA)** **not acceptable (except for TB fat, kg):** VAT, trunk fat (kg, %), TB fat% r = 0.5–0.78; TB fat [kg] r = 0.81 • **Convergent validity of WC (vs. DXA)** **not acceptable (except for trunk fat, kg):** VAT, trunk fat%, TB fat [kg], TB fat% r = 0.47–0.79; trunk fat [kg] r = 0.85
Rankin et al. 2018 [58]	22 (22/0)	Age: 36±10 (18–50) TSI: 8±8 AIS: A-B NLI: 14 para; 8 tetra	• DXA (Lunar Prodigy): trunk fat (kg, %) & LM • MRI: 1.5 T (GE Signa): images of trunk analyzed for LM, SAT, and VAT	• **Convergent validity of DXA trunk (vs. MRI)** **not acceptable**: R^2^ = 0.26–0.29; Bland Altman showed systemic over-estimation of trunk LM of 22.2kg
Ravensbergen et al. 2014 [59]	27 (19/8)	Age: 40±11 TSI: 13.8±9.7 AIS: 20 A-B; 7 C-D NLI: 12 para; 15 tetra	• BMI: mass determined from DXA, length determined with electronic ruler from DXA • WC: supine at narrowest part of waist at end expiration • DXA (Hologic QDR 45000): TB fat (kg, %), abdominal fat (kg, %)	• **Convergent validity of BMI (vs. DXA)** **not acceptable (except for TB fat, kg):** TB % r = 0.73; TB kg r = 0.90 • **Convergent validity of WC (vs. DXA)** **not acceptable**: abdominal fat % r = 0.59; abdominal fat kg r = 0.79
Singh et al. 2014 [60]	95 (71/24)	Age: 33.3 (19–60) TSI: <72hrs AIS: A-B NLI: NR	• BMI: methods NR • DXA (Hologic QDR-2000): TB fat%	• **Convergent validity of BMI (vs. DXA)** **not acceptable**: r = -0.19
Smith et al. 2016 [61]	5 (5/0)	Age: 31±7 (26–44) TSI: NR (1–5 yrs) AIS: C-D NLI: tetra (C5-C8)	• MRI: 3D dual-echo fat-water technique (2-pt Dixon method) to quantify lower leg fat infiltration (gastrocnemius, soleus, tibialis anterior, fibularis longus); measured 1x by 6 technicians	• **Inter-rater reliability (6 raters)** **acceptable**: r = 0.94–0.99
Spungen et al. 1995 [62]	12 (12/0)	Age: 28.5±1.9 (20–38) TSI: 3.8±0.5 (1–6) AIS: NR NLI: tetra (C4-C7)	• BIA (RJL Systems, Model 101A): predict TB FM & FFM • DXA (Lunar Radiation Corp, Model DPX): TB FM & FFM • TBW: measured by the dilution of tritiated water; determine TB FM & FFM • TBK (whole-body ^40^K): determine TB FM & FFM • Skinfolds: biceps, triceps, subscapularis, chest, suprailiac, thorax, umbilicus, abdomen and thigh (Lange calipers and various equations); predict D_b_ and FM & FFM	• **Convergent validity of BIA, DXA, TBW, TBK, skinfolds (vs. mean of all means)** **acceptable**: r = 0.87–0.95 (except for 1 skinfold equation Steinkamp et al r = 0.76)
Spungen et al. 2000 [63]	8 (8/0)	Age: 40±10 (25–58) TSI: 16±9 (3–26) AIS: NR NLI: para (T6-L1)	• BMI: mass measured on scale, length measured supine • DXA (Lunar Radiation Corp, Model DPX,): TB FM	• **Convergent validity of BMI (vs. DXA)** **not acceptable:** r = 0.75
Sumrell et al. 2018 [64]	22 (22/0)	Age: 36±10 (18–50) TSI: 8±8 AIS: AIS A-B NLI: 14 para, 8 tetra	• Anthropometrics (seated & supine): WC (midpoint between crest of ilium and interior margin of last rib) and abdominal circumference (level of umbilicus) at end expiration • MRI: 1.5T (GE): fast echo sequence to obtain 20–30 transverse trunk images for VAT_vol_, VAT_CSA_	• **Convergent validity of anthropometrics (vs. MRI)** **acceptable**: seated/supine WC r = 0.78–0.82; seated/supine abdominal circumference r = 0.79–0.80
Swaine et al. 2018 [65]	16 (8/0)	Age: 31.6±13.6 TSI: 99.6±136.5 mo. AIS: NR NLI: 4 para, 4 tetra	• Ultrasound (Philips, B-Mode) to measure 5 soft tissue layers (total, skin, fat, tendon, muscle) between the lowest point of the ischial tuberosity and overlying skin in loaded & unloaded sitting position; measured 3x by 2 sonographers	• **Intra-rater reliability (3 scans)** **acceptable** **for total, muscle, fat:** ICC_3,1_ = 0.84–0.98 (**not acceptable** **for tendon and skin**: ICC_3,1_ = 0.38–0.65) • **Inter-rater reliability (2 raters)** **acceptable** **for total, muscle, fat**: ICC_2,1_ = 0.80–0.96 (**not acceptable** **for tendon, skin**: ICC_2,1_ = 0.10–0.71)
Wade and Gorgey 2017 [66]	22 (22/0)	Age: 37±10 (18–50) TSI: 8±8 AIS: A-B NLI: 14 para, 8 tetra	• Anthropometrics: thigh circumference and thigh skinfold thickness to determine thigh CSA • MRI: 1.5T (GE Signa): fast spin echo to obtain 12–15 transaxial images of thigh CSA	• **Convergent validity of thigh CSA (vs. MRI CSA)** **acceptable:** R^2^ = 0.90; Bland-Altman showed slight systematic overestimation of anthro thigh CSA
Wade et al. 2018 [67]	22 (22/0)	Age: 37±10 (18–50) TSI: 8±8 AIS: A-B NLI: 14 para; 8 tetra	• Cross validate SCI-specific thigh CSA equation developed in Wade and Gorgey 2017 above • Anthropometrics and MRI same as above	• **Convergent validity of thigh CSA (vs. MRI CSA)** **acceptable**: R^2^ = 0.72; Bland-Altman shows high level of agreement
Wielopolski et al. 2009 [68]	21 (NR)	Age: 51.3±12.0 (23–71) TSI: 12.6±9.7 (1–29) AIS: NR NLI: 10 para; 11 tetra	• PBK: legs body potassium measurement to determine body cell mass and ICW; calculate legs LM • DXA (GE Lunar Prodigy): legs LM	• **Convergent validity of PBK (vs. DXA)** **not acceptable**: R^2^ = 0.32
Willems et al. 2015 [69]	14 (14/0)	Age: 32±7 TSI: 12±7 AIS: A-B NLI: tetra (C5-C7)	• BMI: mass measured on scale, length measured supine • WC: supine at narrowest part of torso at end expiration • Skinfolds: biceps, triceps, subscapular, iliac crest, supraspinale, abdominal, thigh and calf (Harpenden Calipers and various prediction equations); predict TB D_b_ and TB fat% • DXA (GE Lunar Prodigy): TB fat%	• **Convergent validity of BMI & WC (vs. DXA)** **not acceptable**: r = 0.59 & r = 0.62 • **Convergent validity of skinfold (vs. DXA)** **acceptable**: r = 0.87–0.88
Wong et al., 2015 [70]	17 (12/5)	Age: 42.9±10.1 (18–45) TSI: >6mo AIS: A-C NLI: NR	• pQCT (Stratec XCT2000): single image at 66% site of tibia; 2 images obtained and analyzed via watershed (MD & MSCA), threshold-based (MD & MSCA)	• **Test-retest reliability** **inconclusive** **for watershed method**: RMSCV 1.38–1.42%; **inconclusive** **for threshold-based method**: RMSCV 2.94–4.06% (watershed method superior) • **Inter-rater reliability (2 raters)** **inconclusive** **for watershed method:** RMSCV 3.24–3.88%
Yun et al. 2019 [71]	52 (52/0)	Age: 42.1±11.4 TSI: 12±7 AIS: A-B NLI: tetra (C5-C7)	• BMI: mass measured on digital wheelchair scale; length measured supine • WC: supine at the level midway between the lowest rib and iliac crest at end expiration • DXA (GE Lunar Prodigy): TB fat%	• **Convergent validity of BMI (vs. DXA)** **not acceptable**: r = 0.51 • **Convergent validity of WC (vs. DXA)** **not acceptable:** r-0.71

Acronyms (in alphabetical order): ADP = air displacement plethysmography; AIS = American Spinal Injury Association Impairment Scale; BIA = bioelectrical impedance; BIS = bioelectrical spectroscopy; BMI = body mass index; CSA = cross sectional area; CT = computed tomography; D_2_O = deuterium oxide; D_b_ = body density; DXA = dual-energy x-ray absorptiometry; ECV = extracellular volume; ECW = extracellular water; FFM = fat free mass (kg); FM = fat mass (kg); HW = hydrostatic weighing; ICV = intracellular volume; ICW = intracellular water; LM = lean mass (kg); MRI = magnetic resonance imaging; NLI = neurological level of injury; NR = not reported; O_2_ = oxygen; PBK = partial body potassium; SAT = subcutaneous adipose tissue; TB = total body; TOBEC = total body electrical conductivity; TBK = total body potassium; TBW = total body water; TSI = time since injury; US = ultrasound; V_b_ = body volume; VAT_mass_ = visceral adipose tissue mass (kg); VAT_vol_ = visceral adipose tissue transformed to volume using a constant correction factor (density of adipose tissue = 0.94 g/cm^3^); WC = waist circumference.

### Study designs: Measurement properties

The 42 articles that met the eligibility criteria included five studies on test-retest reliability, four studies on intra-rater or inter-rater reliability, 37 studies on convergent validity, and one study on criterion validity (Figs 1 and 2). Studies on other measurement properties, such as responsiveness, were not identified.

### Participant characteristics

As detailed in Table 2, a total number of 1,154 participants were included across the 42 articles, comprised of men (n = 1011) and women (n = 143) aged 16 to 71 years with cervical, thoracic or lumber lesion levels (C1 to L5), complete or incomplete SCI (American Spinal Injury Association Impairment Scale [AIS] A, B, C and D), mostly with a relatively long-standing SCI (all studies included participants ranging from 1 to 36 years post-injury [‘chronic’ SCI], except for three studies [38, 56, 60] including participants ranging from 3 days to 4 months post-injury [‘acute’ SCI]). All studies included SCI-only samples, except for one study that included a sample of at least 50% people with SCI mixed with participants with spinal bifida [32]. In three articles, participants were described as wheelchair athletes [33, 41, 54], while the other articles provided no or limited information about physical activity levels or sports background.

### Body composition assessment tools

Fig 2 shows the 42 studies represented as those evaluating reliability on the left and the same assessment tool showing validity across from it on the right; each assessment tool bubble indicates whether they were whole-body or regional measures. Fig 3 displays in more detail the number of studies reporting acceptable convergent validity for each of the most commonly evaluated assessment tools (i.e., DXA, conductance [e.g., BIA], skinfold thickness, waist circumference, and BMI). Details on each study are provided in Table 2.

#### Whole body composition

A three-compartment model of whole-body composition was assessed using DXA in one test-retest reliability and two convergent validity studies; all three studies reported acceptable reliability [42] or convergent validity [56, 62]. Of note, DXA was used as the reference tool for most of the convergent validity studies on doubly-indirect methods (e.g., conductance, skinfolds, ADP).

A two-compartment model of whole-body composition (total body electrical conductivity) in which participants were positioned in a whole-body cylinder was assessed for its test-retest reliability and was found to be acceptable (55). While no validity studies are available for this conductance method, seven other studies have assessed convergent validity of BIA compared to DXA [34, 41, 54, 62] or total body water [32, 38, 56, 62]; the majority were found to be inconclusive or not acceptable. Another two-compartment model of whole-body composition (skinfold thickness) showed acceptable test-retest reliability [33], however only three of the eight studies assessing whole-body convergent validity of skinfold thicknesses were acceptable when compared to DXA [41, 49, 54, 62, 69], total body water (38, 62), conductance [55, 62], or hydrostatic weighing [33].

Other two-compartment models of whole-body composition (i.e., ADP, total body water, total body potassium) have not been assessed for their reliability; their convergent validity was reported as not acceptable (ADP) [41], not acceptable (total body water) [40] and acceptable (total body water and total body potassium) [62]. One study reported acceptable criterion validity of ADP and hydrostatic weighing when compared to a four-compartment model [36]; however this result of acceptable validity for total body volume was limited to a sample of participants with paraplegia in whom thoracic volume was not obtained and who were able to sit upright without support in the small chamber.

Lastly, the most commonly assessed whole-body composition tool for convergent validity was BMI, although no reliability studies exist. Studies compared BMI to DXA [31, 37, 46, 57, 59, 60, 63, 69, 71] or total body water [32]; none of the studies reported acceptable convergent validity. The consensus from the literature is that BMI values underestimate body fat; in other words, a healthy BMI may mask excessive adiposity in persons with SCI.

#### Regional body composition

Regional body composition (e.g., trunk, thigh, buttocks) was assessed for reliability and convergent validity using DXA, skinfolds, waist circumference, ultrasound, MRI, pQCT, and partial body potassium. While no studies assessed reliability of regional measures using DXA or skinfolds, the majority of articles using DXA to assess convergent validity of regional visceral adipose tissue [50, 58] and thigh lean mass [48, 53] were acceptable. All four studies assessing convergent validity of regional body composition using skinfolds were acceptable [47, 51, 66, 67]; of note, 3 of the 4 studies calculated thigh volume from a combination of skinfold thicknesses and thigh circumference [47, 66, 67]. The most commonly assessed regional body composition tool was waist circumference; one study found acceptable test-retest reliability of waist circumference [8], while four of the 11 validity studies reported acceptable convergent validity [8, 37, 51, 64] of waist circumference compared to DXA [35, 37, 57, 59, 69, 71], CT [8, 45], or MRI [44, 51, 64]. Stronger associations were found when waist circumference was measured supine rather than sitting.

One study using ultrasound assessed inter- and intra-rater reliability of determining the soft tissue layers around the ischial tuberosity and reported some layers to be acceptable and others not acceptable [65]; another study assessing convergent validity of ultrasound for subcutaneous adipose tissue and visceral adipose tissue was not acceptable [39]. A study using MRI to measure subcutaneous adipose tissue and visceral adipose tissue reported inconclusive inter- and intra-rater reliability and not acceptable convergent validity [44]; one other study using MRI to measure thigh/lower leg fat infiltration (i.e., muscle quality) reported acceptable inter-rater reliability [61]. Convergent validity was not acceptable in the one study using partial body potassium to assess legs’ lean mass compared to DXA [68].

Lastly, two studies assessing measurement properties of analysis techniques reported inconclusive test-retest and inter-rater reliability of pQCT-derived watershed and threshold algorithms [70], and acceptable convergent validity of an MRI automated segmentation technique [52].

### Stakeholder consultation

Overviews of the stakeholder consultations with persons with lived SCI and body composition assessment experience as well as with practitioners working in the field of SCI and body composition were summarised (see S2 and S3 Files for details).

The stakeholders with SCI were very clear in their feedback regarding the importance of knowing body composition for their own personal health and empowering their health-related decision making. They felt there was not enough information available on most body composition assessment tools for them to be used with confidence. The stakeholders with SCI considered the measurement burden of DXA acceptable as long as it was reliable, valid and would do no harm (e.g. radiation, skin damage). Practitioners also indicated their confidence that DXA is a reliable and valid assessment tool with acceptable measurement burden for measuring body composition. However, they considered cost and accessibility major barriers, particularly for sport performance settings.

When discussing two-compartment models including conductance, skinfolds, and ADP, opinions and experiences varied. Views on conductance (e.g., BIA) among persons with SCI and the practitioners were mixed and mostly negative. Some of the persons with SCI looked favourably upon the potential of a home-based tool, while others had experienced severe adverse events trying to use it as a standing tool; cost and accessibility were the main barriers identified by the practitioners. Persons with SCI perceived skinfold thicknesses to be a simple tool with relatively little time, effort and equipment required, but were not sure if and how this could be conducted without trained staff. Moreover, practitioners perceived skinfolds to be a reliable tool to monitor intra-individual changes over time, but not for estimating body fat, and noted caution towards skin damage and ensuring correct body positioning. ADP was described as having cost and accessibility barriers by the practitioners. The expert panel noted additional practical barriers based on their experience using ADP among athletes with SCI including participant preparation time, difficulties assessing thoracic volume, and risk of pressure injuries.

Persons with SCI and practitioners had very little confidence regarding the use of BMI and expressed concerns about invalid comparisons to able-bodied normative values. Persons with SCI expressed a preference towards simple tools, but only if they were accurate (e.g., there was little confidence in measuring waist circumference in a seated position). Other less common assessment tools, including ultrasound, MRI, pQCT, body water, and body potassium, were considered difficult to access or cost-prohibitive by the practitioners surveyed. The expert panel regarded many of these tools potentially useful for mechanistic clinical or research studies.

## Discussion

The present scoping review identified a large range of body composition assessment tools and outcomes evaluated in SCI-specific research on measurement properties. Despite the common use of DXA, conductance, skinfolds, waist circumference, and BMI to measure aspects of body composition (e.g., whole-body, regional fat mass or fat-free mass), there is surprisingly little research evidence on test-retest reliability, criterion validity or responsiveness of these tools in the SCI population.

Our review found that whole-body DXA is the only tool with both test-retest reliability [42] and convergent validity [56, 62] evidence to support its use in SCI practice–given that any measurement tool should be both reliable and valid in order to recommend its use in research or clinical practice [30]. However, DXA is costly and inaccessible to many in the field, causing challenges for clinicians, researchers, and other practitioners to obtain reliable and valid body composition measures [11, 13].

Waist circumference and skinfold thickness measurements may hold the most promise as more practical and affordable body composition tools, as evidenced by some of the studies identified in this review [8, 33, 37, 47, 51, 62, 64, 66, 69] and a recently published study [72]. The promise was also highlighted by the views and experiences of the expert panel and the other stakeholders consulted in this study. However, further support is needed for their reliability and validity. Both tools have evidence from a single study reporting acceptable test-retest reliability [8, 33]. They have been commonly researched with regards to convergent validity: 11 studies evaluating waist circumference and 12 studies evaluating skinfold thicknesses against DXA, CT, MRI, or total body water [8, 35, 37, 38, 41, 44, 45, 49, 54, 55, 57, 59, 62, 64, 69, 71]. The majority of these reported statistics that were inconclusive or below threshold values for showing acceptable convergent validity (e.g. minimum ICC of 0.70) [28, 29]. These varied results regarding convergent validity of waist circumference and skinfold thickness measures may be explained by the heterogeneity of the samples included (e.g., injury characteristics), methods of assessment (e.g., waist circumference supine vs sitting, with the latter less likely to be valid), and standardization of measurement conditions (e.g., time of day, before or after exercise, before or after bladder emptying, equipment and software type of the reference method).

Well-established standardization of measurement conditions are of great importance for precision of body composition measurement [73, 74], including that of waist circumference [cf. 72] and skinfold thicknesses [cf. 75]. In addition, it is important to note that many of the body composition assessment tools require a trained professional.

### Research gaps

DXA was used as a reference tool in the majority of studies (19 of the 37 studies) assessing convergent validity of doubly-indirect methods (e.g., conductance, skinfolds, ADP). It should be cautioned, then, that only one study has evaluated the test-retest reliability [42] and two the convergent validity (compared to total body water) of whole-body DXA in persons with SCI [56, 62]. Four other studies have evaluated the convergent validity of regional DXA of the trunk or thigh compared to MRI [48, 50, 53, 58]. Despite the potential of MRI [76], far less SCI-specific evidence was identified for it (e.g., no test-retest reliability studies) than for DXA, possibly due its higher costs and lesser accessibility.

Evidence-based guidelines [22] for proper scan acquisition and analysis in persons with SCI are warranted, as well as more reliability studies using both major commercial manufacturers (GE Medical Systems Inc [formerly Lunar] and Hologic Inc.) [77], and criterion validity studies using a 4-compartment model as a comparator. Such guidelines and studies should also recognize its limitations: obtaining reliable measures in persons with SCI may be challenging due to physical (e.g., contractures, spasticity, hardware in the body, urine reservoirs, obesity, etc.) and logistical (e.g., transferring, positioning) barriers introducing error of measurement [14, 78].

Another important gap identified is that on waist circumference and skinfold measurements. While both waist circumference and skinfold thickness measures are low cost, easy to use, and accessible [51, 59, 64, 66, 72, 75], further research is still required to identify standard techniques, sites, and their reliability, validity, and responsiveness in the SCI population, as indicated by the varying results on convergent validity identified in this review. Notwithstanding, both tools show premise as more practical, accessible and affordable body composition tools [51, 64, 66, 72], particularly for longitudinal tracking within individual subjects [59, 75]. As a caveat, this premise holds for skinfold thickness measures only (if international standards are followed and damaged skin is avoided), rather than any equations that predict percent body fat from skinfolds.

Other important gaps in the literature include the insufficient inclusion of females; only 12% of the sample in the available literature were females, which is much lower than the historical demographics of SCI reported by International Spinal Cord Society [79]. It is possible that some assessment tools may require sex-specific protocols, as highlighted by studies reporting sex dimorphism [31, 54, 80, 81]. Further, most of the studies in the present review involved cohorts with heterogenous injury characteristics (i.e., considerable variation in times since injury, severity of injury, and level of injury); more individualized approaches are needed to identify possible discrepancies in reliability and validity of body composition assessment tools related to injury characteristics.

### Priorities for future research

Overall, our review suggests that the evidence base for reliable and valid assessment of SCI body composition requires further strengthening, and that it is important for practitioners, researchers and people with SCI to continue to work together with this challenge in mind. Accordingly, we formulated a set of priorities and considerations for research in this area (Table 3). These were informed by the available evidence and identified research gaps in the review, and further sensitized by the practical expertise and lived SCI experiences of the expert panel, which were further enriched by the stakeholder workshops and surveys.

**Table 3:** Future priorities and considerations for research on measurement properties of body composition in the SCI population.

1	Establish standardized SCI-specific protocols for assessing body composition via DXA, waist circumference, and skinfold thickness, regarding pre-assessment conditions (e.g., bladder voiding, time of day, exercise and nutrition intake) and recognizing the physical barriers persons with SCI experience (e.g., contractures, spasticity, hardware in the body, urine reservoirs, obesity). These protocols will help reduce measurement error and improve reliability and validity outcomes.
2	Establish reliability over time and across assessors and commercial manufacturers for DXA, waist circumference and skinfold thickness measures. Given that reliability decreases when DXA is performed with special populations (i.e., obese, osteoporotic) [78], it becomes even more important to establish the least significant change (LSC) or smallest detectable difference (SDD) for the SCI population, for example when evaluating serial assessments of nutrition or exercise-induced changes to determine whether a change is real and not due to measurement error.
3	Establish criterion validity of DXA for whole-body composition and regional body composition measures of fat and fat-free mass, using a four-compartment model as the reference method. DXA currently holds the most promise as a reliable and valid imaging technique relatively commonly available in research and clinical settings, but further SCI-specific evidence is needed to endorse it as the surrogate gold standard.
4	Establish responsiveness of waist circumference and skinfold thickness measures as practical, low-cost tools, using a longitudinal design with a comparison to a convergent measure (e.g., DXA site-specific measures) on at least two time points. Along with the reliability studies, this is a prerequisite for obtaining confidence that a change in these measures can be attributed to an intervention and not measurement error.
5	Assess potential sex differences by including females in reliability and validity study designs, and consider the influence of various injury characteristics (e.g., injury level and completeness).

This ambitious research agenda can be facilitated by encouraging robust, standardized data collection through international, multi-center collaboration. Such data collection can provide sufficiently sized samples, allow sub-group analyses (e.g., according to age, sex, training status/physical activity levels), and can substantiate ecological validity (e.g., visceral fat or waist circumference as SCI-specific cardiovascular health indicators, skinfold thickness measures as wheelchair-sport performance indicators). With this dataset, international normative values and clinical practice guidelines for body composition assessment in SCI can be established. It would also provide the foundation for internationally standardized methods and outcome measures for SCI trials such as longitudinal exercise and nutrition trials. As highlighted by others [82] and by our stakeholder consultations, any document guidance must be inclusive of describing any associated risks and accessibility concerns in individuals with SCI.

The panel recommends prioritizing research on the tools described above (i.e., DXA, waist circumference, skinfold thicknesses), rather than techniques that have theoretical flaws for SCI body composition measurement and for which the evidence is currently–at best–inconclusive. Although MRI also holds high promise for reliable and valid assessment in SCI (76), its relative inaccessibility and high cost may continue to limit its use to highly specialized clinical and research studies.

### Strengths and limitations

This is the first systematic scoping review on measurement properties of various tools to assess fat and fat-free mass in adults with SCI, in accordance with international standards [16–18]. The collaborative work of the expert panel ensured the representation of the voices of various stakeholder groups [19, 82], and facilitated setting priorities for future research [83]. The expert panel used a convenience sampling approach to reach out to their professional stakeholder networks. This further stakeholder feedback was only used to sensitize the panel’s views, while remaining aware of potential bias that could occur due to this convenience sampling approach.

For feasibility reasons, the expert panel decided to limit the search for evidence to the most essential measurement properties required to recommend a measurement tool [26]. A wider search including other types of measurement properties (e.g., ecological validity) was piloted, but not considered feasible given the titles and abstracts to scan (n = 10,241), and was unlikely to change the review’s conclusions. The limited reporting in the eligible studies of standardized conditions under which the measurements were conducted (e.g., positioning in the scanner, bladder voiding, position in which participants was sitting/lying, if the same investigator conducted each measurement [73, 74]) surfaced during piloting of the data charting; an attempt to systematically capture these conditions as part of the data charting was deemed infeasible.

### Conclusions

This scoping review has provided more clarity on what SCI-specific evidence does and does not exist for the reliable and valid use of body composition assessment tools. The review can support practitioners and researchers working with people with SCI on assessment of body composition. For the time being, the use of DXA under well-standardized measurement conditions, with awareness of the current limitations in the evidence base, is suggested to practitioners and researchers aiming to assess the effects of nutrition and/or exercise-interventions on whole-body and regional fat- and fat-free mass in adults with SCI. The available evidence and gaps identified in this review affirmed that future research should prioritise reliability and validity studies on: (1) DXA as a surrogate ‘gold standard’ tool to assess whole-body and regional fat and fat-free mass, (2) waist circumference and skinfold thickness measurements as practical low-cost tools to assess whole-body and regional fat mass, and (3) females to explore potential sex differences of body composition assessment tools. Such studies can provide the required evidence to develop normative values and clinical practice guidelines for assessment of body composition in SCI.

## Supporting information

S1 Checklist PRISMA-ScR checklist. (PDF)

S1 File Search strategy each database. (PDF)

S2 File Summary of discussions workshops SCI. (PDF)

S3 File Summary of interactive surveys practitioners. (PDF)
