# Supplementary material for: Assessment of body composition in spinal cord injury: A scoping review
Source: PLoS One. 2021 May 7;16(5):e0251142. doi: 10.1371/journal.pone.0251142 (PMC8104368; doi:10.1371/journal.pone.0251142)
Supplement: S1 File — (PDF) [file pone.0251142.s002.pdf]

Pubmed search strategy

#1	Search (tetraplegia OR paraplegia OR spinal cord injur* OR spinal cord injury OR spinal cord lesion* OR spinal cord transection* OR spinal cord impairm* OR spinal injur* OR spinal injury OR spinal lesion* OR spinal transection* OR spinal impairm* OR spine injur* OR spine injury OR spine lesion* OR spine transection* OR spine impairm* OR brown-sequard's syndrome OR brown-sequard OR brown-sequard syndrome OR brown-sequard's OR cauda equina syndrome* OR central cord syndrome* OR anterior cord syndrome* OR posterior cord syndrome*)
#2	Search SCI[Text Word]
#3	Search "myelitis" OR "myelopathy" OR "spinal cord diseases"
#4	Search myelitis[MeSH Terms]
#5	Search "spinal cord diseases"[MeSH Terms]
#6	Search ("spinal" AND "cord" AND "diseases")
#7	Search (body composition OR body constitution OR muscle OR muscle mass OR lean mass OR lean body mass OR lean tissue mass OR fat OR fat free mass OR fat-free mass OR FFM OR LBM OR skeletal muscle OR lean soft tissue OR fat mass OR body fat OR FM OR adipose tissue OR adipose OR adiposity OR non-adipose tissue OR regional adiposity OR adipose tissue distribution OR visceral adiposity OR truncal adiposity OR truncal obesity OR trunk adiposity OR trunk fat OR trunk obesity OR abdominal fat OR abdominal lipid OR intra-abdominal OR visceral fat OR ectopic fat OR liver fat OR central fat OR central fat distribution OR central obesity OR centrally-distributed fat OR centrally-distributed obesity OR visceral obesity OR tissue fat OR subcutaneous fat OR body mass OR body weight OR bodyweight OR total body weight OR total bodyweight OR total body mass OR total body water OR TBW OR obesity OR sarcopenia OR myopenia)
#8	Search (MRI OR fMRI OR MRS OR NMR OR magnetic resonance OR magnetic resonance imaging OR functional magnetic resonance imaging OR magnetic resonance spectroscopy OR CT OR computed tomography OR computerised tomography OR computerized tomography OR computer-tomography OR peripheral quantitative computed tomography OR peripheral quantitative computed tomography OR waist circumference OR WC OR waist-to-hip ratio OR WHR OR waist-to-height ratio OR WHtR OR waist to stature ratio OR conicity index OR BIA OR bioelectrical impedance analysis OR bioelectrical impedance OR electric impedance OR electric resistance OR DEXA OR DXA OR dual-energy x-ray absorptiometry OR dual-energy x-ray absorbtiometry OR dual-energy x-ray absorptiometry OR dual-energy x-ray OR dual x-ray absorptiometry OR dual x-ray absorbtiometry OR dual x-ray absorptiometry OR dual x-ray OR air displacement plethysmography OR ADP OR whole-body air displacement plethysmography OR hydrostatic weighing OR underwater weighing OR hydrostatic body composition analysis OR hydrodensitometry OR hydro-static weighing OR under-water weighing OR hydro-static body composition analysis OR hydro-densitometry OR UWW OR isotope dilution analysis OR isotope dilution OR hydrogen isotope dilution OR hydrogen dilution OR deuterium isotope dilution OR deuterium dilution OR skinfold OR skinfolds OR skinfold measurement OR skinfold thickness OR skin fold OR skin folds OR skin fold measurement OR skin fold thickness)
#9	Search ((#1 OR #2 OR #3 OR #4 OR #5 OR #6) AND #7 AND #8)

Cochrane search strategy

#1	tetraplegia or paraplegia or spinal cord injury or spinal cord lesion or spinal cord transection or spinal cord impairment or spinal injury or spinal lesion or spinal impairment or spine injury or spine lesion or spine transection or spine impairment or brown-sequard's syndrome or brown-sequard or brown-sequard syndrome or brown-sequard's or cauda equina syndrome or central cord syndrome or anterior cord syndrome or posterior cord syndrome or myelitis or spinal cord diseases or myelopathy or SCI
#2	MeSH descriptor: [Myelitis] explode all trees
#3	MeSH descriptor: [Spinal Cord Diseases] explode all trees
#4	("spinal" and "cord" and "diseases")
#5	body composition or body constitution or muscle or muscle mass or lean mass or lean body mass or lean tissue mass or fat or fat free mass or fat-free mass or FFM or LBM or skeletal muscle or lean soft tissue or fat mass or body fat or FM or adipose tissue or adipose or adiposity or non-adipose tissue or regional adiposity or adipose tissue distribution or visceral adiposity or truncal adiposity or truncal obesity or trunk adiposity or trunk fat or trunk obesity or abdominal fat or abdominal lipid or intra-abdominal or visceral fat or ectopic fat or liver fat or central fat or central fat distribution or central obesity or centrally-distributed fat or centrally-distributed obesity or visceral obesity or tissue fat or subcutaneous fat or body mass or body weight or bodyweight or total body weight or total bodyweight or total body mass or total body water or TBW or obesity or sarcopenia or myopenia
#6	MRI or fMRI or MRS or NMR or magnetic resonance or magnetic resonance imaging or functional magnetic resonance imaging or magnetic resonance spectroscopy or CT or computed tomography or computerised tomography or computerized tomography or computer-tomography or peripheral quantitative computed tomography or peripheral quantitative computed tomography or waist circumference or WC or waist-to-hip ratio or WHR or waist-to-height ratio or WHtR or waist to stature ratio or conicity index or BIA or bioelectrical impedance analysis or bioelectrical impedance or electric impedance or electric resistance or DEXA or DXA or dual-energy x-ray absorptiometry or dual-energy x-ray absorbtiometry or dual-energy x-ray absorptiometry or dual-energy x-ray or dual x-ray absorptiometry or dual x-ray absorbtiometry or dual x-ray absorptiometry or dual x-ray or air displacement plethysmography or ADP or whole-body air displacement plethysmography or hydrostatic weighing or underwater weighing or hydrostatic body composition analysis or hydrodensitometry or hydro-static weighing or under-water weighing or hydro-static body composition analysis or hydro-densitometry or UWW or isotope dilution analysis or isotope dilution or hydrogen isotope dilution or hydrogen dilution or deuterium isotope dilution or deuterium dilution or skinfold or skinfolds or skinfold measurement or skinfold thickness or skin fold or skin folds or skin fold measurement or skin fold thickness
#7	(#1 OR #2 OR #3 OR #4) AND #5 AND #6

Embase (OVID) search strategy

#1	(tetraplegia or paraplegia or spinal cord injur* or spinal cord injury or spinal cord lesion* or spinal cord transection* or spinal cord impairm* or spinal injur* or spinal injury or spinal lesion* or spinal transection* or spinal impairm* or spine injur* or spine injury or spine lesion* or spine transection* or spine impairm* or brown-sequard's syndrome or brown-sequard or brown-sequard syndrome or brown-sequard's or cauda equina syndrome* or central cord syndrome* or anterior cord syndrome* or posterior cord syndrome*).mp. [mp=title, abstract, heading word, drug trade name, original title, device manufacturer, drug manufacturer, device trade name, keyword, floating subheading word, candidate term word]
#2	SCI.mp. [mp=title, abstract, heading word, drug trade name, original title, device manufacturer, drug manufacturer, device trade name, keyword, floating subheading word, candidate term word]
#3	"myelitis" or "myelopathy" or "spinal cord diseases").mp. [mp=title, abstract, heading word, drug trade name, original title, device manufacturer, drug manufacturer, device trade name, keyword, floating subheading word, candidate term word]
#4	Myelitis/ (“map term to subject heading”)
#5	Spinal cord disease/ (“map term to subject heading”)
#6	("spinal" and "cord" and "diseases").mp. [mp=title, abstract, heading word, drug trade name, original title, device manufacturer, drug manufacturer, device trade name, keyword, floating subheading word, candidate term word]
#7	(body composition or body constitution or muscle or muscle mass or lean mass or lean body mass or lean tissue mass or fat or fat free mass or fat-free mass or FFM or LBM or skeletal muscle or lean soft tissue or fat mass or body fat or FM or adipose tissue or adipose or adiposity or non-adipose tissue or regional adiposity or adipose tissue distribution or visceral adiposity or truncal adiposity or truncal obesity or trunk adiposity or trunk fat or trunk obesity or abdominal fat or abdominal lipid or intra-abdominal or visceral fat or ectopic fat or liver fat or central fat or central fat distribution or central obesity or centrally-distributed fat or centrally-distributed obesity or visceral obesity or tissue fat or subcutaneous fat or body mass or body weight or bodyweight or total body weight or total bodyweight or total body mass or total body water or TBW or obesity or sarcopenia or myopenia).mp. [mp=title, abstract, heading word, drug trade name, original title, device manufacturer, drug manufacturer, device trade name, keyword, floating subheading word, candidate term word]
#8	(MRI or fMRI or MRS or NMR or magnetic resonance or magnetic resonance imaging or functional magnetic resonance imaging or magnetic resonance spectroscopy or CT or computed tomography or computerised tomography or computerized tomography or computer-tomography or peripheral quantitative computed tomography or peripheral quantitative computed tomography or waist circumference or WC or waist-to-hip ratio or WHR or waist-to-height ratio or WHtR or waist to stature ratio or conicity index or BIA or bioelectrical impedance analysis or bioelectrical impedance or electric impedance or electric resistance or DEXA or DXA or dual-energy x-ray absorptiometry or dual-energy x-ray absorbtiometry or dual-energy x-ray absorptiometry or dual-energy x-ray or dual x-ray absorptiometry or dual x-ray absorbtiometry or dual x-ray absorptiometry or dual x-ray or air displacement plethysmography or ADP or whole-body air displacement plethysmography or hydrostatic weighing or underwater weighing or hydrostatic body composition analysis or hydrodensitometry or hydro-static weighing or under-water weighing or hydro-static body composition analysis or hydro-densitometry or UWW or isotope dilution analysis or isotope dilution or hydrogen isotope dilution or hydrogen dilution or deuterium isotope dilution or deuterium dilution or skinfold or skinfolds or skinfold measurement or skinfold thickness or skin fold or skin folds or skin fold measurement or skin fold thickness).mp. [mp=title, abstract, heading word, drug trade name, original title, device manufacturer, drug manufacturer, device trade name, keyword, floating subheading word, candidate term word]
#9	1 OR 2 OR 3 OR 4 OR 5 OR 6
#10	7 AND 8 AND 9
