# Supplementary material for: Assessment of body composition in spinal cord injury: A scoping review
Source: PLoS One. 2021 May 7;16(5):e0251142. doi: 10.1371/journal.pone.0251142 (PMC8104368; doi:10.1371/journal.pone.0251142)
Supplement: S2 File — (PDF) [file pone.0251142.s003.pdf]

Summary of discussions during workshops with people with lived SCI and body composition assessment experience

	Body mass/ BMI	Skinfold Callipers	BIA	Waist circumference	Hydrostatic Weighing	DXA
Preferences	Use my scale at home, to see if I have gained weight.	Nice and simple, if they were accurate.	I love the BIA because it is so simple. More adaptable, anything at home.		If it is accessible. Even underwater, I would not care. Especially if it is most accurate.	DXA, it was fine. They do DXA here all the time. It's fast and easy. No problems with skin.<br>Over a period of time, for both bone and fat.
Barriers	You don't get weighed a lot when you are in a wheelchair.	I did not even bother with my legs.	The scales [BIA] did not work.<br>Break a bone when measuring on BIA with a standing frame.<br>Obviously that one is out.	My waistline has always been too big. While sitting now, it's just bigger.	Not discussed	I can't even get on DXA bed as it's so much higher than my chair.<br>Inconvenience is making the appointment, put you on the time, etc.<br>I get a lot of pain when I am lying flat.<br>If a DXA scan has metal, it does not work.<br>Radiation is a concern.
Comments	When I weigh myself, it is all fat?					Do the DXA scores go in the same pool as the able-bodied population?

General Comments	- Very important.
	- Predict a lot about potential health. Tells us something how different we are from the able-bodied population. Good for medical populations.
	- Over long term would be helpful. Knowing where you stand.
	- Consistent measure even if it was not accurate.
	- There's just nothing.
	- As long as it is reliable and accurate, I feel that most people I think would go for accurate over convenience.
	- Anything would be helpful.
	- Probably not interested in fat mass; just use weight.
	- I'd like to know. If it's SCI specific.
	- Affects how you want to change your lifestyle (eating, exercise).
	- You need a more specific guideline to go along with an accurate measure.
	- If it was causing harm then I would not do that.
	- Only if it was really important for monitoring health.
	- I think you start obsessing on it over. That's with everything with health-related stuff.
	- I don't see any problems, as long as it is valid. Considering body type, genetics, diet.
	- Just focussing on that [parameters], you can't even enjoy your body anymore.
	- You need people who understand paralysis. For example. It's about vigilance. If you have a C4 who is overweight, how do you really help that person?
