# Supplementary material for: Assessment of body composition in spinal cord injury: A scoping review
Source: PLoS One. 2021 May 7;16(5):e0251142. doi: 10.1371/journal.pone.0251142 (PMC8104368; doi:10.1371/journal.pone.0251142)
Supplement: S3 File — (PDF) [file pone.0251142.s004.pdf]

Summary of interactive surveys of international practitioners working in the field of body composition for SCI (e.g. SCI clinicians, nutritionists, physiologists)

	Body mass/ BMI	Skinfold Callipers	BIA	Waist circumference	ADP	DXA
Preferences	BMI - Not recommended to be used.	Mixed views on using this method.	Unsure to use this method.	Undecided on using this method.	Mixed views on using this method.	Very confident with using this method.
Barriers	Really don't think we should be using BMI given it only gives you mass, nothing else	Hard to standardise seated position when taking. Leads to difficulties getting to sites.<br>When using skinfolds, I do not use equations for calculating % BF. As a repeatable measure within specific athletes I think it is a good measure.<br>Positional difficulties.<br>Athletes desire to measure skinfolds on lower limbs (unsure of accuracy and also worried about potential damage to skin).	Cost and access to kit.	Not discussed	Cost and access to kit.	Ethical considerations for signing off a DXA in a sport-setting as a tracking tool.<br>Consistent placement of body during DXA.<br>Cost and access to kit.

General Comments	- Educational tool, can be helpful to prevent problems we are currently seeing, e.g. ordering a wheelchair but people not fitting in it anymore after it arrives (i.e. 3 months later) due to fat mass increases.<br>- Muscle mass more important than fat mass, because fat mass will vary so much more across life, while muscle mass less so and therefore may be a better indicator of CV risk.<br>- Depends on the relationship with CV markers and such, if/how I consider reliability/validity.<br>- Depends on what you're using it for, tracking changes over time or something else.
